# Exploratory Grouping Along a Shared Oral-Health Burden Continuum in Children and Adolescents: An Age-Stratified Cluster Analysis

**DOI:** 10.3390/medicina62091654

**Published:** 2026-08-28

**Authors:** Narcis Mihaita Bugala, Smaranda-Adelina Bugala, Mihaela Jana Țuculină, Ancuta Ramona Camen, Alina Nicoleta Capitanescu, Dragos Cadea, Adrian Macovei, Loredana Selaru, Ana Maria Rica, Dana Maria Albulescu

**Affiliations:** 1 Doctoral School, University of Medicine and Pharmacy of Craiova, 200349 Craiova, Romania; bugala.mihai@yahoo.com; 2 Department of Endodontics, Faculty of Dental Medicine, University of Medicine and Pharmacy of Craiova, 200349 Craiova, Romania; r_ana_maria22@yahoo.com; 3 Department of Occupational Medicine, Faculty of Medicine, University of Medicine and Pharmacy of Craiova, 200349 Craiova, Romania; ancutzaboicea@gmail.com; 4 Department of Otorhinolaryngology, Faculty of Medicine, University of Medicine and Pharmacy of Craiova, 200349 Craiova, Romania; alinacapitanescu@yahoo.com; 5 Department of Anatomy, Faculty of Medicine, University of Medicine and Pharmacy of Craiova, 200349 Craiova, Romania; dragos.cadea@umfcv.ro (D.C.); adrian.macovei@umfcv.ro (A.M.); med73danam@yahoo.com (D.M.A.); 6 Department of Pediatrics, Faculty of Medicine, University of Medicine and Pharmacy of Craiova, 200349 Craiova, Romania; loredana.selaru@gmail.com

**Keywords:** dental caries, cluster analysis, dental plaque index, periodontal index, gingivitis, child, adolescent, cross-sectional studies, oral health

## Abstract

*Background and Objectives*: Caries experience, plaque accumulation, gingival inflammation, and periodontal screening findings may coexist as manifestations of a shared oral-health burden. We examined whether these routinely recorded indicators form distinct multivariable profiles or primarily represent ordered levels along a common burden continuum in children and adolescents. *Materials and Methods*: This secondary exploratory analysis included 638 participants from a multicenter cross-sectional clinical database: 407 aged 6–12 years and 231 aged 13–19 years. Permanent-dentition Decayed, Missing, and Filled Teeth score, Plaque Index, Gingival Index, and Community Periodontal Index were standardized and analyzed separately by age stratum. Unsupervised K-means clustering compared solutions containing two to five groups to summarize participant-level patterns without imposing predefined clinical categories. Hierarchical Ward classification assessed algorithmic concordance; principal component analysis and a composite standardized burden score assessed dimensionality. Sensitivity analyses excluded the periodontal index or treated it as ordinal with Gower-distance partitioning around medoids, and 1000 repeated 80% subsamples assessed sampling stability using the adjusted Rand index. *Results*: A single principal component explained 91.0% of variance at 6–12 years and 91.8% at 13–19 years, with all four indicators loading strongly on the same dimension. Ordered cluster membership correlated closely with the composite burden score (Spearman ρ = 0.942 and 0.939; both *p* < 0.001). The retained low-, intermediate-, and high-burden groupings were highly consistent when the periodontal index was excluded or treated as ordinal and under repeated subsampling. In adolescents, a two-group alternative preserved the low- and high-burden extremes while dividing the intermediate group, indicating that the number of groups changes descriptive granularity rather than revealing a separate phenotype. *Conclusions*: The principal new finding is that four commonly used oral-health indicators converge on one dominant participant-level burden dimension rather than defining distinct clinical phenotypes. This supports an integrated epidemiological description of cumulative oral-health burden while cautioning against interpreting data-driven groups as diagnostic or treatment categories. The numerical group distributions are sample-specific and require external and longitudinal validation before being transferred to other populations or clinical decision-making.

## 1. Introduction

Oral diseases remain highly prevalent and unequally distributed worldwide, with dental caries representing one of the most common chronic conditions across childhood and adolescence [1,2,3,4,5,6]. Conventional epidemiological reporting usually presents caries experience, plaque accumulation, gingival inflammation, and periodontal screening findings as separate outcomes. Although this approach is necessary for disease-specific surveillance, it may not capture the way multiple clinical indicators coexist within the same participant [6,7,8,9,10,11,12].

Pediatric oral-health surveys have also shown that caries experience rarely occurs independently of oral-hygiene conditions and broader behavioral or social exposures. Romanian studies have reported substantial regional variation in caries prevalence, treatment experience, oral-hygiene practices, and gingival status among schoolchildren and adolescents [13,14,15,16,17,18,19]. These findings support the need to examine several clinical indicators jointly, particularly in settings where access to preventive and restorative care may differ according to geographical and socioeconomic circumstances. However, most available Romanian investigations have analyzed caries, plaque accumulation, or gingival inflammation as separate outcomes rather than as components of a multivariable burden structure.

Caries and plaque-induced gingival conditions are clinically distinct, but they share several determinants related to biofilm accumulation, oral-hygiene behavior, diet, fluoride exposure, and access to dental care [20,21,22,23,24,25,26]. Cross-sectional surveys in pediatric and adolescent populations have repeatedly identified parallel gradients in plaque, gingival inflammation, and caries experience, although the magnitude of these relationships varies between populations and measurement protocols [23,24,25,26]. A multivariable profiling approach may therefore provide a complementary description of how these indicators coexist within individual participants, provided that the resulting groups are not interpreted as causal, diagnostic, or pathophysiological categories.

Data-driven clustering can summarize participants with similar multivariable patterns without imposing predefined diagnostic categories [27,28,29,30,31,32,33]. Depending on the covariance structure of the included variables, clustering may reveal qualitatively different combinations of findings or may partition participants into ordered levels along a common severity dimension. The latter result is particularly likely when the clustering variables are strongly concordant. Cluster-derived groups must therefore be interpreted according to the observed structure of their centers rather than assumed to represent distinct biological or clinical phenotypes. Because clustering is sensitive to variable scaling, distance, the selected number of groups, and the algorithm used, transparent comparison of candidate solutions and cautious interpretation are required [27,29,30,31,32,33].

Association and regression analyses quantify relationships between individual indices, whereas clustering asks a different descriptive question: whether the joint values recorded in the same participant form recurrent multivariable configurations or mainly partition a shared severity continuum. This distinction is relevant when strongly related clinical indicators are considered together, because an apparently well-separated cluster solution may still reflect thresholds imposed on one dominant latent dimension rather than qualitatively different profiles. Compared with separate index-by-index reporting, this participant-level approach can show whether unfavorable findings accumulate within the same individuals and can summarize that co-occurrence for integrated epidemiological surveillance and hypothesis generation. It does not replace disease-specific assessment or establish clinical risk categories.

Accordingly, the present analysis was designed to complement, rather than replicate, the companion CPI–DMF-T association analysis by describing the joint multivariable structure of DMF-T, PI, GI, and CPI and by explicitly distinguishing empirical burden groupings from diagnostic or biological phenotypes.

The primary objective was to explore oral-health burden groupings separately among participants aged 6–12 years and 13–19 years using permanent-dentition DMF-T, CPI, GI, and PI. The secondary objectives were (1) to compare candidate K-means solutions and assess concordance with a Ward hierarchical classification derived from the same variables, and (2) to examine whether group membership varied according to age, sex, area of residence, or county. Additional revision-stage analyses evaluated whether the retained groupings primarily reflected one common severity dimension and whether they remained robust to alternative CPI handling, an alternative adolescent k = 2 solution, and repeated subsampling.

## 2. Materials and Methods

### 2.1. Study Design, Setting, and Relationship to the Companion Analysis

This observational multicenter cross-sectional clinical study was conducted between April 2025 and April 2026 in Southwest Romania. Recruitment occurred in four dental care settings: one school dental office in Gorj County, one school dental office in Olt County, and two centers in Dolj County, comprising one school dental office and the Endodontics Clinic of the Faculty of Dentistry, University of Medicine and Pharmacy of Craiova. These locations served as recruitment and examination settings; irrespective of recruitment site, all standardized study examinations were performed by the same trained examiner described in Section 2.5, rather than by separate site-specific examiners.

The present report is a secondary exploratory analysis of the same 638-participant database used in a companion manuscript examining age-stratified associations between CPI categories and permanent-dentition DMF-T. Recruitment, eligibility criteria, clinical measurements, examiner calibration, and data-quality procedures are therefore shared by the two reports. The companion analysis estimates bivariate and adjusted associations between CPI and DMF-T, whereas the present analysis evaluates the joint multivariable burden structure of DMF-T, PI, GI, and CPI through unsupervised clustering. No additional participants, alternative clinical definitions, or independent validation sample were introduced. The companion manuscript is disclosed to the journal and will be supplied to the editors together with information regarding its current submission status, thereby permitting assessment of methodological overlap and the incremental scientific contribution of the present analysis.

The present study is part of a broader research project conducted under a single ethics-approved protocol. The overarching research protocol was structured around three distinct, predefined research directions, each addressing different research questions and analytical objectives. Accordingly, the same ethical approval applies to the studies and manuscripts derived from these three research directions because the data were collected within the same approved overarching protocol. Although the studies originate from the same research protocol, each manuscript addresses a distinct research direction and analytical objective, and no results are duplicated across publications.

The study was reported in accordance with the Strengthening the Reporting of Observational Studies in Epidemiology (STROBE) recommendations for cross-sectional studies [34]. A completed STROBE checklist is intended to accompany the revised submission as non-published Supplementary Materials. The companion manuscript is disclosed to the journal and will be supplied separately to the handling editor to facilitate assessment of methodological overlap and incremental contribution.

### 2.2. Participants, Recruitment, and Eligibility

Patients attending the participating dental facilities for examination or treatment during the study period were screened consecutively. A consecutive non-probability sampling method was used. All patients presenting during recruitment were assessed for eligibility and invited to participate when the predefined criteria were met. No recruitment quotas or fixed county-level targets were used; the near-equal final county totals occurred incidentally. Because recruitment was facility-based and non-probabilistic, the analytical sample was not designed to be population-representative. Accordingly, prevalence estimates, cluster proportions, and numerical cluster centers should be interpreted as characteristics of this clinical sample rather than direct estimates for all children and adolescents in Southwest Romania.

Participants were eligible if they were 6–19 years old, attended one of the participating facilities during the recruitment period, had at least one CPI-eligible sextant containing a minimum of two fully erupted and clinically assessable permanent teeth, and could undergo the standardized oral examination. Only permanent teeth contributed to all study indices.

Exclusion criteria were fixed orthodontic treatment; severe dento-maxillary anomalies preventing standardized examination; acute oral conditions requiring immediate treatment before completion of the study examination; systemic diseases or medication use known to substantially alter gingival or periodontal findings, including abnormal bleeding, marked immune dysfunction, or gingival enlargement; professional periodontal treatment or systemic antibiotic use within the previous three months; inability to cooperate; and incomplete clinical records or missing data for the principal variables.

A total of 674 individuals were assessed. Thirty-six were excluded because one or more eligibility criteria were not met, and 638 were included. The original screening records did not preserve mutually exclusive counts for each exclusion reason; this limitation is reported transparently. The analytical sample was divided a priori into participants aged 6–12 years (*n* = 407) and 13–19 years (*n* = 231).

### 2.3. Study Size

The parent clinical study used a pragmatic sample-size calculation intended to provide reasonable precision for oral-health prevalence estimates rather than formal power for cluster analysis. At a 95% confidence level, an expected proportion of 50%, and an absolute precision of 4%, the estimated minimum was 600 participants. Allowing approximately 5% for incomplete or unusable records produced a target of at least 630; the final analytical sample was 638.

No separate formal sample-size formula was applied to determine the number of clusters. Candidate solutions were evaluated within each age stratum using internal validation indices, cluster sizes, clinical coherence, and an alternative clustering algorithm.

### 2.4. Clinical Variables and Measurement

#### 2.4.1. Permanent-Dentition Caries Experience

Dental caries experience was assessed exclusively in the permanent dentition using the Decayed, Missing, and Filled Teeth index, reported as DMF-T, according to World Health Organization criteria [35]. Primary teeth were not included, and no lowercase dmft variable was calculated or analyzed.

Only fully erupted permanent teeth that could be examined reliably were included. Partially erupted permanent teeth and teeth that could not be assessed adequately were excluded from scoring. Caries was diagnosed by visual-tactile examination under artificial light with a plane dental mirror and a blunt-ended probe (E. HAHNENKRATT GmbH, Königsbach-Stein, Germany); radiographs were not used. Teeth missing for reasons unrelated to caries, including orthodontic extraction, trauma, or congenital absence, were not counted in the M component.

#### 2.4.2. Community Periodontal Index

A full-mouth CPI-based screening protocol adapted from World Health Organization principles was applied to fully erupted and clinically assessable permanent teeth [35,36]. Six sites per eligible tooth were examined with a WHO periodontal probe (E. HAHNENKRATT GmbH, Königsbach-Stein, Germany). A sextant was eligible when it contained at least two fully erupted assessable permanent teeth. Third molars, primary teeth, exfoliating teeth, and partially erupted permanent teeth were excluded.

For each eligible sextant, the highest finding was recorded: CPI 0, no bleeding on probing and no detectable calculus; CPI 1, bleeding on probing without detectable calculus; and CPI 2, supra- or subgingival calculus. No CPI 3 or 4 findings were recorded. Participant-level CPI was defined as the highest score recorded across all eligible sextants. For the cluster analysis, CPI was coded as an ordered numeric feature with values of 0, 1, and 2. This coding preserved the clinical order of the observed categories but treated adjacent categories as equally spaced when Euclidean distances were calculated. CPI was therefore interpreted as an ordinal screening component of the multivariable burden structure rather than as a continuous periodontal measurement.

#### 2.4.3. Plaque Index and Gingival Index

Plaque accumulation was measured using the Silness-Löe Plaque Index and gingival inflammation using the Löe-Silness Gingival Index [37,38]. Distal, facial or buccal, mesial, and lingual or palatal surfaces of each fully erupted and assessable permanent tooth were scored from 0 to 3 according to the original criteria. Tooth-level means were calculated first, followed by participant-level means across all eligible permanent teeth.

PI and GI were calculated as participant-level continuous means from the eligible fully erupted permanent teeth examined according to the standardized protocol. Their role as clustering features, together with the coding and analytical handling of the remaining variables, is described in Section 2.7.

### 2.5. Examiner Calibration and Data Quality

All examinations were performed by a single examiner trained in the DMF-T, CPI, PI, and GI protocols. Intra-examiner agreement was assessed by repeating the complete oral examination in a randomly selected subsample of 62 participants after 7–10 days, without access to the initial findings. Agreement across the available categorical clinical recordings was summarized using a global Cohen’s kappa coefficient of 0.86. Separate repeated-measure records suitable for retrospective index-specific reanalysis were not available for the present revision. Accordingly, the global coefficient is retained only as a general indicator of examiner consistency and is not presented as evidence that DMF-T counts, ordinal CPI categories, or continuous participant-level PI and GI means had equivalent index-specific reliability.

Clinical forms were checked for completeness before database entry. The database was screened for duplicate records, values outside permitted ranges, internal inconsistencies, and coding errors. Participants were represented by unique numerical study codes, and directly identifying information was excluded from the analytical dataset.

### 2.6. Bias and Missing Data

Consecutive screening and uniform eligibility criteria were used to reduce discretionary selection within the participating facilities. Examination by one trained examiner eliminated inter-examiner variation, although index-specific intra-examiner reliability estimates were not available. Age-stratified analysis was specified to reduce heterogeneity related to dental development. Nevertheless, non-probability recruitment from clinical settings creates selection bias and limits population representativeness.

There were no missing values for age, sex, residence, county, DMF-T, PI, GI, or CPI among the 638 included participants. The cluster analyses therefore used complete cases without imputation. Participants with incomplete principal data had been excluded at the eligibility/data-quality stage.

### 2.7. Statistical Analysis

Statistical analyses were performed using JASP (Version 0.96.0; JASP Team, University of Amsterdam, Amsterdam, The Netherlands) [39]. Tests were two-sided, and *p* < 0.05 was considered statistically significant for external comparisons. Analyses were performed separately in the 6–12-year and 13–19-year strata. Additional sensitivity and resampling calculations requiring explicit Gower dissimilarities, PAM clustering, and adjusted Rand index computations were executed through R version 4.5.2 in JASP using the same complete-case data.

Within each age stratum, DMF-T, PI, GI, and numerically coded CPI were standardized to z scores so that the four clustering features contributed on comparable scales. K-means clustering with Euclidean distance was used as an exploratory unsupervised method because the study question concerned whether participants formed recurrent multivariable configurations without imposing predefined diagnostic or risk categories. DMF-T, PI, and GI were quantitative participant-level measures, whereas CPI was an ordinal screening variable coded 0, 1, and 2. Consequently, its inclusion in Euclidean clustering assumed equal numerical spacing between CPI 0 and CPI 1 and between CPI 1 and CPI 2. This assumption was accepted for the primary exploratory analysis to preserve the ordered clinical information contained in CPI, but it should not be interpreted as evidence that the clinical differences between consecutive CPI categories are quantitatively equivalent. Candidate solutions with k = 2, 3, 4, and 5 were compared to determine whether a parsimonious partition could summarize the observed joint structure without producing poorly separated or very small groups. Explained standardized variance (R^2^) quantified the proportion of standardized variation captured by a partition; the silhouette coefficient quantified within-group cohesion relative to between-group separation; and the Calinski–Harabasz index contrasted between-group with within-group dispersion. Akaike information criterion (AIC) and Bayesian information criterion (BIC) were used as complementary relative-fit measures available in the JASP K-means implementation and were compared only among candidate solutions fitted to the same standardized variables within each age stratum. Because information criteria may favor more complex partitions, they were interpreted jointly with separation indices, group size, and clinical coherence rather than as stand-alone evidence of an optimal number of groups [27,28,29,30,39].

Principal component analysis (PCA) was performed separately in each age stratum to answer a distinct question from clustering: whether the covariance among DMF-T, PI, GI, and CPI was dominated by one common severity dimension or required several independent dimensions. For the PCA, CPI was entered using the same ordered numerical coding (0, 1, and 2) as in the primary K-means analysis. Component retention was evaluated by parallel analysis. A simple composite burden score was also calculated as the arithmetic mean of the four standardized z scores to provide an interpretable continuous reference for the same joint burden. Because K-means labels are arbitrary, the retained clusters were recoded in clinically ordered form (1 = low, 2 = intermediate, 3 = high burden), and Spearman’s rho was used to quantify the monotonic correspondence between ordered group membership and the continuous composite score without assuming equal spacing between the three cluster labels.

Candidate K-means solutions were evaluated jointly according to statistical separation, explained variance, group size, and clinical coherence. Clinical coherence was used only as a descriptive interpretability criterion after the candidate solutions had been inspected; it was not a prespecified validation endpoint. A solution was considered clinically coherent when its centers showed an internally interpretable ordering across the four indices without producing implausibly small groups. In the 6–12-year stratum, k = 3 was retained because it produced the highest silhouette coefficient and Calinski–Harabasz index. In the 13–19-year stratum, the selection criteria were discordant: k = 2 produced the highest silhouette coefficient, whereas k = 3 produced the highest Calinski–Harabasz index, explained a more standardized variance, and separated an intermediate-burden group. The three-group solution was retained for descriptive resolution, while k = 2 was treated as an explicit alternative solution rather than as an inferior model. For interpretation, qualitatively distinct multivariable patterns would require non-parallel standardized center profiles, with different indices preferentially elevated in different groups. Conversely, broadly parallel monotonic shifts across all four indicators were interpreted as partitioning of a shared burden continuum, particularly when supported by a dominant principal component and close correspondence with a composite burden score. This interpretive framework was descriptive and was not used as an additional model-selection criterion.

The retained groups were labeled comparatively low, intermediate, or high burden after examination of their standardized centers and observed clinical values. These labels were descriptive and relative to the distributions within each age stratum. They were not prespecified diagnostic categories or validated clinical thresholds. Because DMF-T, PI, GI, and CPI were the variables used to construct the groups, their subsequent gradients across the retained classifications were summarized descriptively and were not treated as independent evidence validating the groups.

Operational differentiation of burden levels was cluster-derived rather than based on prespecified clinical cut-offs. Within each age stratum, participants were assigned by K-means to the nearest centroid in the four-dimensional standardized DMF-T, PI, GI, and CPI space. The three retained clusters were then ordered according to their centroid profiles and observed clinical values and labeled comparatively low, intermediate, and high burden. Thus, the effective thresholds were the multivariable decision boundaries equidistant between adjacent cluster centroids; no single DMF-T, PI, GI, CPI, or composite-score threshold defined group membership. These labels are sample-relative descriptive categories and should not be interpreted as validated diagnostic or treatment thresholds.

Age was compared across the retained groups using the Kruskal–Wallis test because the post-clustering comparison concerned differences in the distribution of a continuous variable across three independent groups without relying on normality; rank epsilon squared was reported as an effect-size measure. One-way ANOVA was retained as a complementary descriptive analysis. Associations of group membership with sex, area of residence, and county were assessed using chi-square tests because these variables were categorical, with Cramer’s V used to summarize association strength. These post-clustering demographic comparisons were exploratory and descriptive; no multiplicity correction was applied, and non-significant findings were not interpreted as evidence of demographic, geographical, or service-level homogeneity. Recruitment-center identifiers were not retained as an analytical covariate, so center effects could not be evaluated separately from county.

Robustness to the CPI specification was examined in two additional ways. First, K-means clustering with k = 3 was repeated using DMF-T, PI, and GI only. Second, Gower dissimilarity was calculated with CPI treated as an ordered variable and partitioning around medoids (PAM) was fitted with k = 3. Alternative cluster labels were aligned by low–intermediate–high burden ordering before participant-level agreement was summarized; adjusted Rand index (ARI) was additionally calculated for the Gower-PAM comparison. Because silhouette favored k = 2 in adolescents, the complete 13–19-year two-cluster solution was characterized using standardized centers, observed DMF-T/PI/GI values, CPI 0/1/2 distributions, demographics, and cross-classification against the three-cluster solution. Sampling stability of the retained k = 3 partitions was then assessed by 1000 repeated subsamples containing 80% of participants without replacement. The reference partitions were reproduced with Hartigan–Wong K-means using random seed 1, 100 random starts, a maximum of 100 iterations, and the algorithm’s standard convergence rule; each subsample used 50 random starts and the same iteration limit. Stability was summarized by the ARI distribution, including its mean, median, dispersion, empirical 2.5–97.5th percentiles, and the proportion of repetitions with ARI ≥ 0.90. Details of these revision-stage analyses are provided in Supplementary Tables S1–S4.

### 2.8. Ethics Approval and Consent

The study was conducted in accordance with the Declaration of Helsinki and approved by the Ethics Committee of the University of Medicine and Pharmacy of Craiova, Romania (No. 143/28 March 2025). Written informed consent was obtained from parents or legal guardians for participants younger than 18 years, together with age-appropriate assent where applicable. Participants aged 18 years or older provided their own written informed consent. The analysis used de-identified clinical data.

## 3. Results

### 3.1. Participant Flow

**Figure 1:**
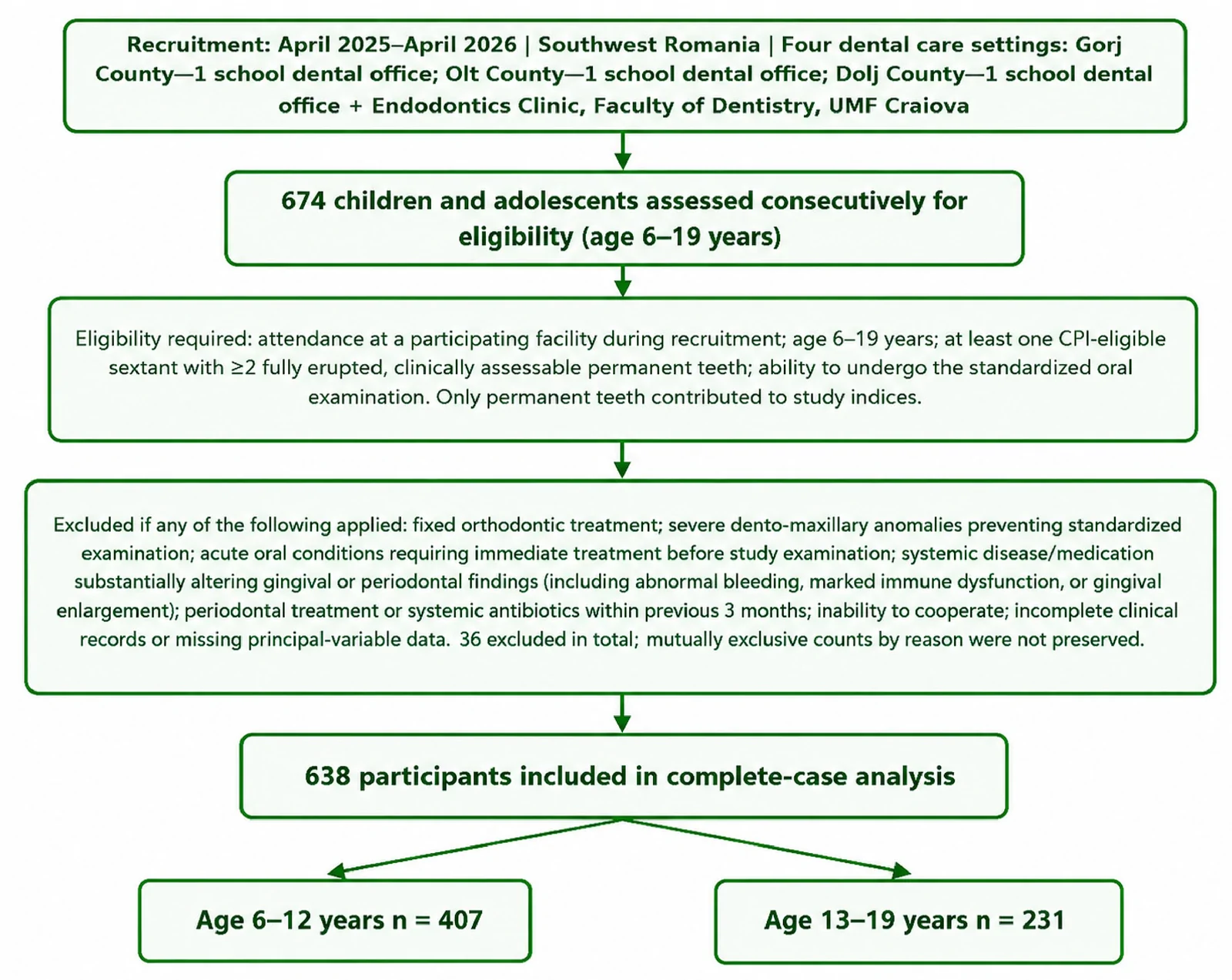
Participant flow through eligibility assessment and age-stratified analysis. Mutually exclusive numerical counts for individual exclusion reasons were not available. Recruitment period, setting, detailed eligibility criteria, and exclusion criteria are shown explicitly in the revised flow diagram.

Between April 2025 and April 2026, 674 children and adolescents were assessed for eligibility across the four participating dental care settings. Thirty-six individuals (5.3%) were excluded because at least one predefined eligibility criterion was not met. Mutually exclusive numerical counts for individual exclusion reasons were not available. The final complete-case sample comprised 638 participants (94.7% of those assessed), including 407 participants aged 6–12 years and 231 aged 13–19 years. All included participants had complete data for the demographic variables and the four clustering indicators. The participant flow is summarized in Figure 1.

### 3.2. Participant Characteristics

The 6–12-year stratum included 407 participants with a mean age of 9.64 ± 1.78 years; 218 (53.6%) were girls, and 245 (60.2%) lived in urban areas. Mean permanent-dentition DMF-T was 4.71 ± 2.55, mean PI was 1.78 ± 0.79, and mean GI was 1.35 ± 0.72. The 13–19-year stratum included 231 participants with a mean age of 14.64 ± 1.52 years; 119 (51.5%) were girls, and 128 (55.4%) lived in urban areas. Mean DMF-T was 4.93 ± 2.58, mean PI was 1.84 ± 0.85, and mean GI was 1.38 ± 0.73 (Table 1).

### 3.3. Evaluation of Candidate K-Means Solutions

In the younger stratum, the information criteria decreased as the number of groups increased, with the lowest AIC and BIC values observed for k = 5. However, k = 3 produced the highest silhouette coefficient (0.530) and Calinski–Harabasz index (932.6) and explained 82.2% of the standardized variance. Increasing k to 4 or 5 produced lower silhouette and Calinski–Harabasz values and divided the sample into smaller, more fragmented groups. The three-group solution was therefore retained because it provided the best separation according to both the silhouette and Calinski–Harabasz indices while preserving non-trivial group sizes, despite the lower AIC and BIC values of the more complex solutions.

**Figure 2:**
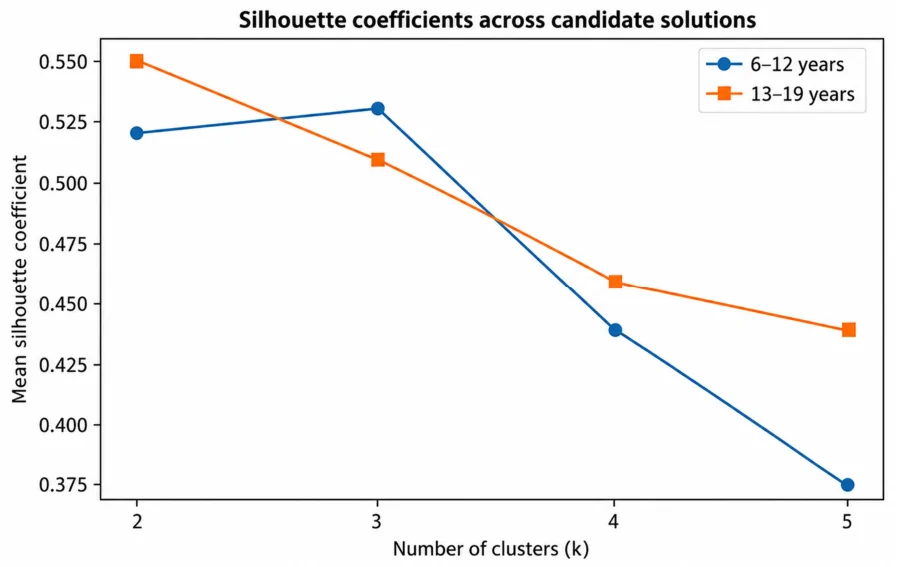
Silhouette coefficients for candidate K-means solutions in the two age strata.

**Figure 3:**
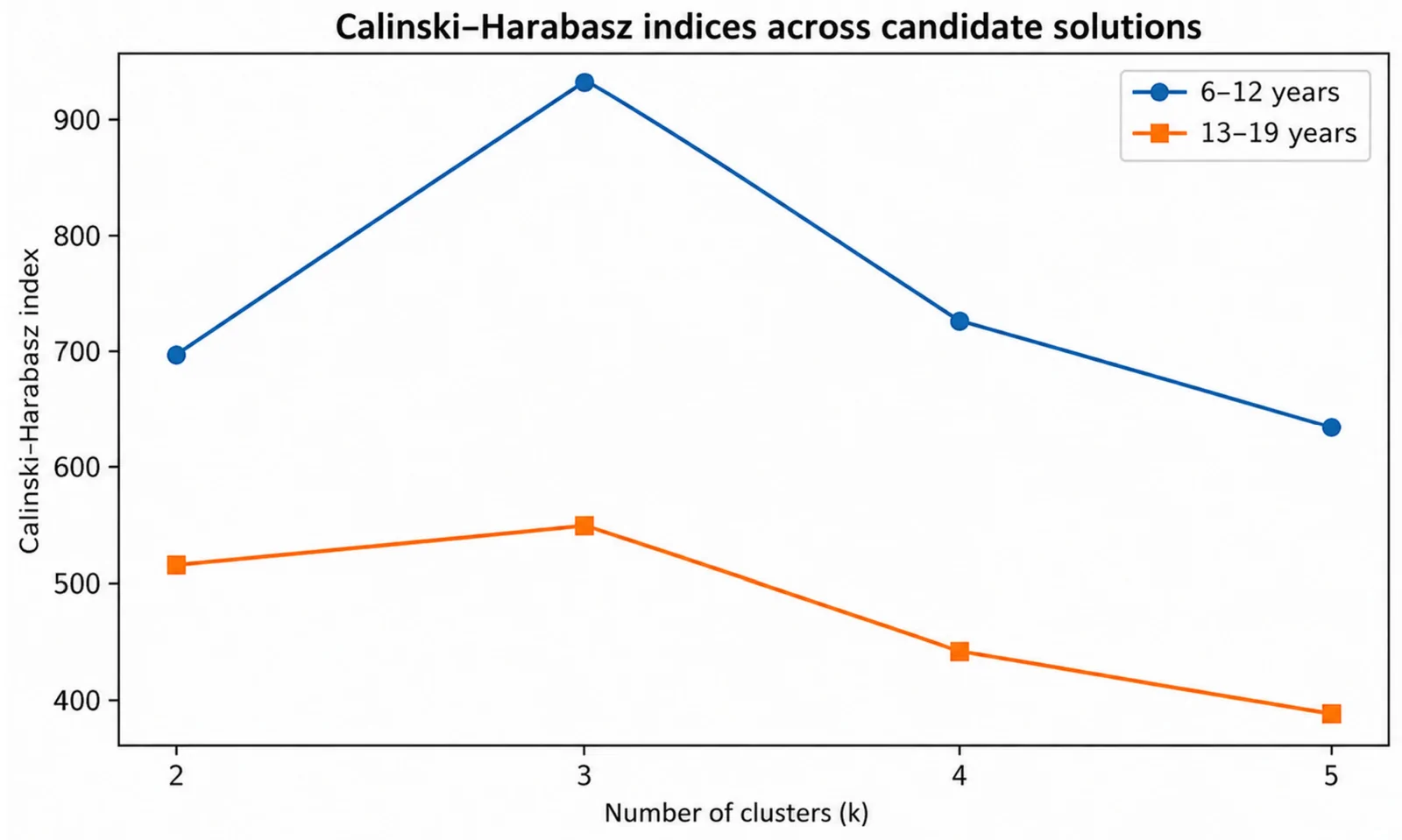
Calinski-Harabasz indices for candidate K-means solutions in the two age strata.

In the older stratum, the model-selection criteria favored different solutions. The two-group solution had the highest silhouette coefficient (0.550), indicating the strongest average cohesion and separation. The three-group solution had the highest Calinski–Harabasz index (549.4) and increased R^2^ from 0.692 to 0.828. AIC continued to decrease through k = 5, whereas BIC reached its minimum at k = 4. However, the four- and five-group solutions had lower silhouette and Calinski–Harabasz values and included several relatively small groups. The three-group solution was retained as a balance between explained variance, group separation, group size, and descriptive clinical resolution, but neither the information criteria nor the remaining internal indices identified it as uniquely optimal (Table 2; Figure 2 and Figure 3).

### 3.4. Exploratory Burden Groups in Participants Aged 6–12 Years

**Figure 4:**
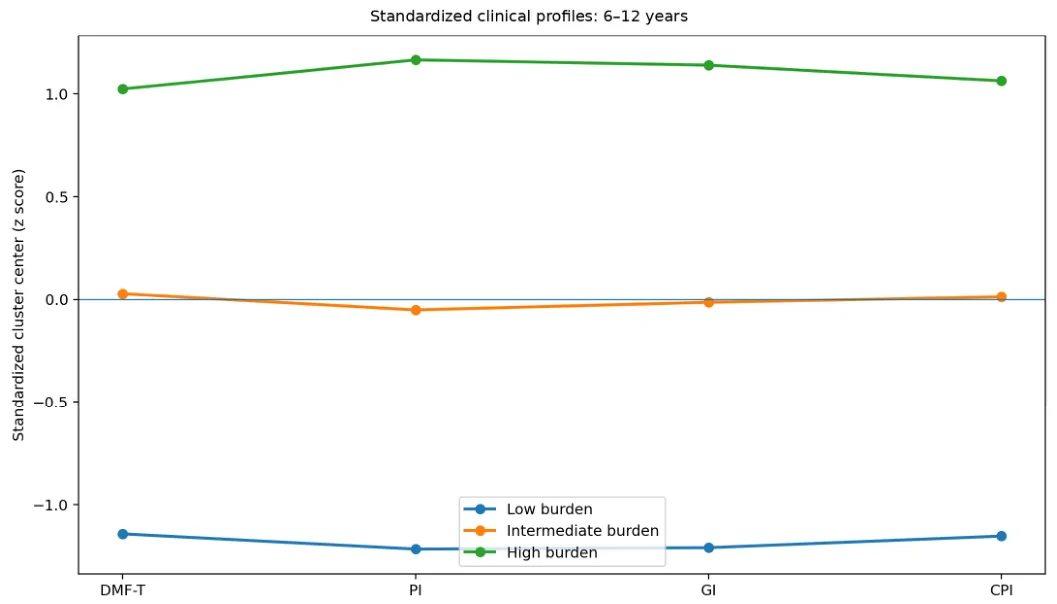
Standardized centers of the retained three-cluster solution among participants aged 6–12 years.

The retained solution separated 123 participants (30.2%), 151 (37.1%), and 133 (32.7%) into comparatively low-, intermediate-, and high-burden groups. Mean permanent-dentition DMF-T increased from 1.80 to 4.78 and 7.32, respectively, with corresponding increases in PI, GI, and CPI. Thus, the retained classification primarily partitioned participants according to overall indicator magnitude rather than identifying discordant combinations of caries, plaque, gingival inflammation, and CPI findings. Because the same variables defined and characterized the groups, these gradients are descriptive features of the partition and not independent validation of clinically distinct categories (Figure 4; Table 3).

### 3.5. Exploratory Burden Groups in Participants Aged 13–19 Years

**Figure 5:**
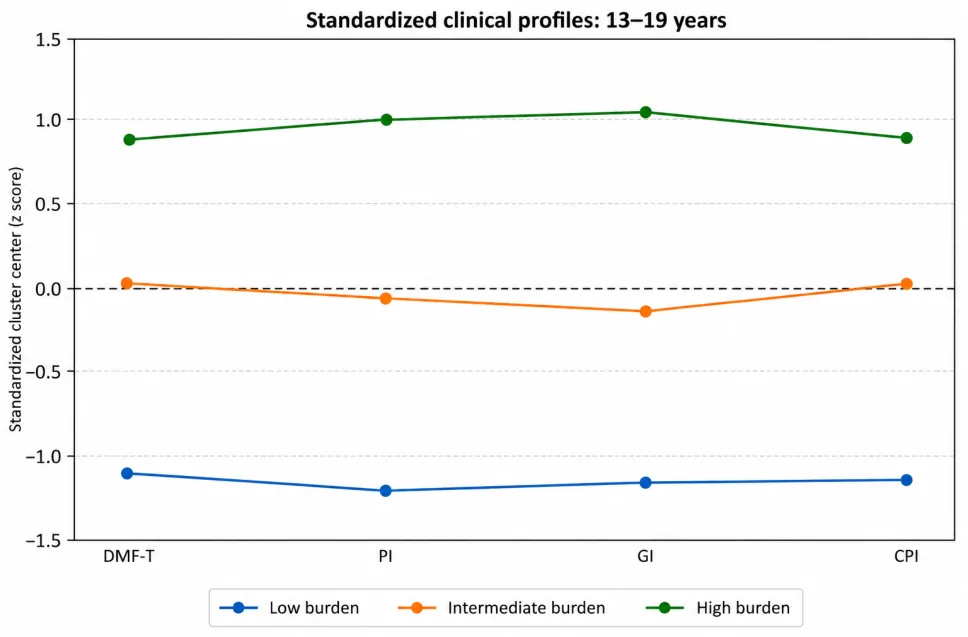
Standardized centers of the retained three-cluster solution among participants aged 13–19 years.

The exploratory three-group solution separated 76 participants (32.9%), 63 (27.3%), and 92 (39.8%) into comparatively low-, intermediate-, and high-burden groups. Mean DMF-T increased from 2.05 to 4.97 and 7.28, with corresponding increases in PI, GI, and CPI. CPI 0 was recorded in 92.1% of the low-burden group, CPI 1 in 74.6% of the intermediate-burden group, and CPI 2 in 79.3% of the high-burden group. The close alignment between CPI categories and the retained groups indicates an ordered severity continuum rather than qualitatively different oral-health patterns. As in the younger stratum, these gradients arise from variables used to construct the groups and therefore should not be interpreted as independent clinical validation (Figure 5; Table 3).

### 3.6. Dimensionality, Sensitivity, Alternative Adolescent Solution, and Sampling Stability

PCA demonstrated a dominant common dimension in both age strata. Among participants aged 6–12 years, the first component had an eigenvalue of 3.640 and explained 91.0% of total variance; loadings were 0.924 for DMF-T, 0.975 for PI, 0.959 for GI, and 0.958 for CPI. Among participants aged 13–19 years, the first component had an eigenvalue of 3.672 and explained 91.8% of variance, with loadings of 0.930, 0.977, 0.962, and 0.962, respectively. Parallel analysis retained one component in each stratum. The composite burden score closely tracked the ordered K-means classifications (Spearman ρ = 0.942 for 6–12 years and ρ = 0.939 for 13–19 years; both *p* < 0.001), supporting interpretation of the clusters as empirical partitions along a common burden continuum (Supplementary Table S1).

Sensitivity analyses indicated that the low–intermediate–high structure did not depend materially on the numerical inclusion of CPI. When CPI was excluded and K-means was repeated using DMF-T, PI, and GI only, 392/407 participants aged 6–12 years (96.3%; Cramer’s V = 0.947) and 222/231 participants aged 13–19 years (96.1%; Cramer’s V = 0.939) retained the equivalent burden assignment after label alignment. Gower-distance PAM with CPI treated as ordinal yielded corresponding three-level burden structures and agreed with the original K-means labels for 396/407 younger participants (97.3%; ARI = 0.918) and 220/231 adolescents (95.2%; ARI = 0.866). Reclassifications occurred almost entirely between adjacent burden levels rather than directly between low and high burden (Supplementary Table S2).

The alternative adolescent k = 2 solution comprised 105 lower-burden and 126 higher-burden participants and had the highest silhouette coefficient among the adolescent candidate solutions (0.550). Standardized centers were uniformly lower in cluster 1 (DMF-T −0.914, GI −0.906, PI −0.943, CPI −0.873) and higher in cluster 2 (0.762, 0.755, 0.786, and 0.728, respectively). Observed mean DMF-T was 2.57 versus 6.90, mean PI 1.04 versus 2.50, and mean GI 0.72 versus 1.93. CPI 0/1/2 distributions were 74/30/1 and 2/42/82. The original k = 3 low-burden group mapped entirely to the lower-burden k = 2 group and the original high-burden group entirely to the higher-burden group, whereas the 63-member intermediate group split almost evenly (29 vs. 34). This pattern indicates that the third group adds descriptive granularity within a broad severity continuum rather than identifying an isolated phenotype (Supplementary Table S3).

Repeated 80% subsampling confirmed high within-sample stability of the retained k = 3 partitions. Across 1000 repetitions, mean ARI was 0.996 in the 6–12-year stratum (median 1.000; empirical 2.5–97.5th percentile 0.990–1.000; minimum 0.971) and 0.987 in the 13–19-year stratum (median 0.985; empirical 2.5–97.5th percentile 0.968–1.000; minimum 0.926). All repetitions in both strata had ARI ≥ 0.90. These results demonstrate strong stability to the tested subsampling perturbation, although they do not substitute for validation in an independent cohort (Supplementary Table S4).

### 3.7. Exploratory Demographic Distribution Across the Retained Burden Groups

In the 6–12-year stratum, age did not differ across the retained groups (Kruskal–Wallis H = 0.195, *p* = 0.907, rank epsilon squared < 0.001). Group membership was not associated with sex (chi-square = 3.240, *p* = 0.198, Cramer’s V = 0.089), residence (chi-square = 0.951, *p* = 0.621, Cramer’s V = 0.048), or county (chi-square = 1.451, *p* = 0.835, Cramer’s V = 0.042).

In the 13–19-year stratum, age also did not differ significantly (H = 4.100, *p* = 0.129, rank epsilon squared = 0.018). No significant associations were observed with sex (chi-square = 2.727, *p* = 0.256, Cramer’s V = 0.109), residence (chi-square = 1.361, *p* = 0.506, Cramer’s V = 0.077), or county (chi-square = 2.857, *p* = 0.582, Cramer’s V = 0.079) (Table 4). These comparisons were exploratory and were not multiplicity-adjusted. Because recruitment-center identifiers were not available as a separate analytical variable, the absence of county associations should not be interpreted as evidence that school and university-clinic recruitment settings were homogeneous.

### 3.8. Concordance Between K-Means and Ward Classifications

When a three-group Ward hierarchical classification was imposed on the same standardized variables, the resulting groups followed a low–intermediate–high ordering similar to that observed with K-means. Among participants aged 6–12 years, the Ward classification had R^2^ = 0.818 and a silhouette coefficient of 0.520, with group sizes of 128, 151, and 128. After the Ward groups were matched to the K-means groups according to their standardized cluster centers, 395 of 407 participants (97.1%) received the same burden label, with Cramer’s V = 0.957.

Among participants aged 13–19 years, the Ward classification had R^2^ = 0.827 and a silhouette coefficient of 0.510, with group sizes of 90, 76, and 65. After matching the groups according to their standardized cluster centers, Ward and K-means burden labels agreed for 227 of 231 participants (98.3%), with Cramer’s V = 0.973. These results demonstrate high concordance between the two algorithms when applied to the same variables and participants. They do not evaluate the stability of group membership under repeated sampling or establish reproducibility in an independent population (Table 5).

## 4. Discussion

### 4.1. Principal Findings and Relationship to the Companion Analysis

The central knowledge gained from this analysis is not simply that three numerical groups can be generated, but that four routinely recorded oral-health measures converge on one dominant participant-level burden dimension. Although the clinical sample could be separated into ordered low-, intermediate-, and high-burden groups in both age strata, the first principal component explained 91.0% of variance in the 6–12-year stratum and 91.8% in the 13–19-year stratum, with strong loadings for all four variables. The composite mean z-score also correlated very strongly with ordered cluster membership (ρ = 0.942 and 0.939). Thus, the apparent groups are best understood as stable empirical segments of a shared oral-health burden continuum rather than evidence of distinct pathophysiological phenotypes. This distinction is the principal interpretive contribution of the study.

The novelty of the present analysis lies in testing this continuum interpretation directly rather than assuming that a well-separated clustering solution represents biologically distinct subtypes. The companion study estimates age-stratified associations between CPI and DMF-T and examines how those relationships change after adjustment for PI or GI. Here, the same routinely collected indicators are evaluated jointly at the participant level, their dimensional structure is quantified, alternative partitions are compared, and robustness is challenged by removing CPI, treating CPI as ordinal through Gower distance, changing the clustering algorithm, and repeatedly perturbing the observed sample. The persistence of the low-to-high ordering across these analytical choices shows that the main signal is cumulative burden shared across the four indices, not an artifact of one clustering specification. The contribution is therefore a multivariable epidemiological description of how unfavorable findings accumulate within the same individuals, not a new causal mechanism or diagnostic classification system. Previous studies in adolescent populations have likewise documented substantial burdens of dental caries and periodontal health problems, supporting the relevance of considering these oral-health dimensions jointly [40,41].

From a clinical and public-health perspective, this finding suggests that permanent-dentition caries experience, plaque accumulation, gingival inflammation, and periodontal screening findings should not always be viewed as isolated survey outcomes: in this sample they largely moved together along the same burden axis. This may support integrated descriptive surveillance and inform future prevention-oriented research by identifying participants in whom several unfavorable oral findings accumulate simultaneously. At the same time, the study does not establish diagnostic thresholds, prognosis, treatment response, or the clinical benefit of assigning an individual to a burden group. The low-, intermediate-, and high-burden labels are therefore descriptive summaries of relative position within the observed sample, not validated risk or treatment categories [7,8,9,10,11,12,42,43,44,45].

### 4.2. Interpretation of Cluster Selection and Algorithmic Concordance

Model selection still requires caution, especially in adolescents. In the 6–12-year stratum, silhouette and Calinski–Harabasz supported k = 3, whereas AIC and BIC favored more complex partitions. In the 13–19-year stratum, silhouette supported k = 2, Calinski–Harabasz supported k = 3, BIC supported k = 4, and AIC supported k = 5. The explicit k = 2 sensitivity analysis clarifies the practical consequence of this discordance: the original low- and high-burden adolescent groups remained intact, while the intermediate group was divided almost evenly between the two broader groups. Thus, k = 3 provides descriptive granularity along the continuum, whereas k = 2 provides a coarser partition with stronger silhouette separation. Neither representation should be described as uniquely correct.

The dimensionality and CPI sensitivity analyses further explain why multiple cluster criteria can support different partitions. PCA showed that more than 90% of variance in each stratum was captured by one component, so different values of k primarily impose different cut-points along a strong common axis. Importantly, the substantive low-to-high ordering remained highly consistent when CPI was excluded and when Gower dissimilarity treated CPI as ordinal. Participant-level agreement with the original K-means classification remained above 95% in every sensitivity analysis, and Gower-PAM ARI values were 0.918 and 0.866. These findings reduce concern that the three-level structure was created solely by equal-spacing coding of CPI, while still acknowledging that any partition of a near-unidimensional continuum depends on analytical choices [46,47,48,49].

Agreement between K-means and Ward exceeded 97% in both age strata and should be interpreted as algorithmic concordance using the same data and Euclidean framework. The new repeated-subsampling analysis addresses a different question: whether the retained K-means assignments remain stable when the observed sample is perturbed. Mean ARI values were 0.996 and 0.987 across 1000 80% subsamples, and every repetition in both strata exceeded ARI 0.90. This provides strong evidence of internal sampling stability within the present dataset. It does not, however, demonstrate transportability to other populations, centers, or measurement settings; independent external replication remains necessary.

Age, sex, area of residence, and county were not significantly associated with retained group membership. These analyses were exploratory, multiple post-clustering comparisons were performed without formal multiplicity correction, and the non-significant results should not be interpreted as evidence of demographic, geographical, or service-level homogeneity. Recruitment center could not be analyzed separately from county, and the database did not include detailed measures of diet, fluoride exposure, household resources, dental attendance, or parental education [2,7,50,51]. The observed clinical co-occurrence therefore cannot be attributed empirically to any specific behavioral or socioeconomic determinant.

The retained classifications are therefore most useful as a descriptive epidemiological summary. Their potential value lies in showing that multiple routine oral-health indices can identify participants who accumulate several unfavorable findings simultaneously, which may help motivate integrated preventive assessment and hypothesis generation. At the same time, because the four variables used to define the groups are strongly concordant and largely unidimensional, the low/intermediate/high labels should not be presented as independent phenotypes, validated risk classes, or decision thresholds. More clinically differentiated subtypes may require behavioral, dietary, socioeconomic, preventive-care, microbiological, or longitudinal variables that were not available here [52,53].

The present study does not demonstrate that the three-group representation is clinically or inferentially superior to treating burden as a continuous score; its added value is limited to a pragmatic descriptive summary of relative burden segments for communication, tabulation, and exploratory surveillance. Accordingly, the terms low, intermediate, and high refer to observed burden within this sample and are not synonymous with low-, intermediate-, or high-risk categories.

### 4.3. Strengths and Limitations

Strengths include transparent use of the same recruitment and measurement framework as the companion analysis, a priori age stratification, consistent use of permanent-dentition DMF-T in both strata, simultaneous integration of four routine clinical measures, comparison of k = 2–5 solutions, explicit reporting of discordant model-selection criteria, and acknowledgment that the adolescent three-group solution is not uniquely optimal. The revision adds direct dimensionality assessment, a composite burden comparison, complete presentation of the adolescent k = 2 alternative, sensitivity analyses excluding CPI and using ordinal Gower-PAM clustering, participant-level agreement metrics, and repeated subsampling stability assessment. Consecutive screening, examination by one trained examiner, complete principal variables, and documented participant flow also improve reporting transparency.

Several limitations should be considered. First, the cross-sectional design prevents assessment of temporal sequence, transitions between burden groups, or prediction of future disease. Second, consecutive non-probability recruitment from dental care settings introduces selection bias, and the sample should not be considered representative of all children and adolescents in Southwest Romania. The detailed mutually exclusive reasons for the 36 exclusions were not retained.

Third, only fully erupted permanent teeth were assessed. Particularly among participants aged 6–12 years, the number of erupted and examined permanent teeth and the number of CPI-eligible sextants may have differed substantially according to dentition stage. These quantities were not retained as separate analytical variables and could not be recovered for the present revision. Consequently, no denominator-based DMF-T sensitivity analysis or direct comparison of examined-tooth/sextant counts across burden groups could be performed. Participants may therefore have differed in their opportunity to accumulate DMF-T components and in the number of teeth or sites available for PI, GI, and CPI assessment. Age stratification reduced but could not eliminate this source of heterogeneity, and chronological age is not a complete proxy for dentition stage. The study does not quantify total caries burden across both dentitions in younger participants. Fourth, DMF-T is cumulative, its D, M, and F components were not analyzed separately, and radiographs were not used.

Fifth, CPI is an ordinal screening indicator rather than a continuous periodontal measurement or comprehensive pediatric periodontal diagnosis. Coding CPI as 0, 1, and 2 in the primary Euclidean K-means analysis imposes equal numerical spacing between adjacent categories, which is not necessarily clinically equivalent. The revision directly tested this concern: the low–intermediate–high ordering remained highly consistent when CPI was removed and when CPI was treated as ordinal using Gower-distance PAM, with participant-level agreement above 95%. These results support robustness to the tested specifications but do not make the original numerical assumption intrinsically correct. Sixth, PCA confirmed that the four clustering variables are strongly concordant and largely represent a single dimension. The retained groups should therefore be understood as reproducible numerical partitions of that burden continuum rather than independently validated multivariable phenotypes. The same variables used to construct the groups cannot independently validate them, and internal resampling stability does not replace external validation.

Seventh, recruitment center was not retained as a separate analytical covariate, so differences between school dental offices and the university clinic could not be evaluated independently of county and may have influenced the observed distributions. Behavioral, socioeconomic, microbiological, and treatment-history variables were unavailable. Eighth, intra-examiner agreement was summarized using a single global Cohen’s kappa coefficient derived from categorical clinical recordings. The underlying repeated-examination records required for index-specific weighted kappa/ICC reanalysis were not available for this revision; accordingly, the global coefficient is retained only as a general indicator of examiner consistency and cannot establish equivalent reproducibility for DMF-T, CPI, PI, and GI. Finally, use of the same dataset in two manuscripts requires clear editorial disclosure; the companion manuscript will be supplied separately to the editors so that overlap and incremental contribution can be assessed directly. Residual measurement inconsistency could attenuate cluster separation or shift participants near empirical cut-points between adjacent burden groups; therefore, the observed clustering and resampling stability should be interpreted in the context of this measurement limitation.

### 4.4. Generalizability and Future Research

The transferability of the present findings should be separated into two components. The numerical group proportions, cluster centers, and empirical cut-points are directly applicable only to this consecutively recruited clinical sample and measurement protocol and should not be generalized as population estimates for Southwest Romania or other settings. In contrast, the broader structural finding—that several routinely collected caries, plaque, gingival, and periodontal screening indicators may align predominantly along one common burden dimension—is a testable hypothesis that may be transferable to other pediatric populations. Confirmation requires independent community-based and clinical cohorts, other Romanian regions and countries, and settings with different preventive and treatment access. External replication should therefore evaluate both the dimensional structure itself and the extent to which numerical group boundaries and prevalence vary across populations.

Future research should determine whether these highly stable within-sample burden partitions transport to independent population-based cohorts and longitudinal settings. Replication should include center-level sampling information and explicit dentition-stage denominators in younger children. Studies integrating behavioral, dietary, fluoride-exposure, socioeconomic, preventive-care, microbiological, and treatment-history variables may determine whether clinically differentiated multivariable phenotypes emerge beyond the single dominant burden dimension observed here. Longitudinal data are also required to test transitions between burden levels, prognostic relevance, and whether any empirically derived grouping adds value to preventive or therapeutic decision-making.

## 5. Conclusions

This study shows that the clinically familiar pattern of concurrent caries experience, plaque accumulation, gingival inflammation, and periodontal screening findings can be summarized by one dominant participant-level oral-health burden dimension. The low-, intermediate-, and high-burden groupings were highly stable across alternative handling of CPI, an alternative clustering framework, and repeated subsampling, indicating that the underlying low-to-high gradient is robust within this dataset. The important implication is therefore not the creation of three new phenotypes, but the demonstration that several routine indices largely converge on a common cumulative-burden signal that can be described jointly for epidemiological surveillance and hypothesis generation. In adolescents, the two-group alternative preserved the low and high extremes while dividing the intermediate group, reinforcing that the number of groups mainly changes descriptive resolution along the same continuum. Because recruitment was non-probabilistic and facility-based, the numerical partitions should not be transferred directly to other populations. Independent population-based and longitudinal studies are required to determine whether the shared burden dimension replicates across settings and whether any derived grouping provides prognostic or clinical decision-making value.

## Figures and Tables

**Table 1:** Participant characteristics by age stratum.

Characteristic	6–12 Years (*n* = 407)	13–19 Years (*n* = 231)
Age, years, mean ± SD	9.64 ± 1.78	14.64 ± 1.52
Girls, *n* (%)	218 (53.6)	119 (51.5)
Urban residence, *n* (%)	245 (60.2)	128 (55.4)
Dolj/Gorj/Olt, *n*	131/146/130	82/67/82
DMF-T, mean ± SD	4.71 ± 2.55	4.93 ± 2.58
PI, mean ± SD	1.78 ± 0.79	1.84 ± 0.85
GI, mean ± SD	1.35 ± 0.72	1.38 ± 0.73
CPI 0/1/2, *n* (%)	127 (31.2)/156 (38.3)/124 (30.5)	76 (32.9)/72 (31.2)/83 (35.9)

Note. DMF-T was assessed only in fully erupted permanent teeth in both strata. CPI, Community Periodontal Index; DMF-T, Decayed, Missing, and Filled Teeth; GI, Gingival Index; PI, Plaque Index; SD, standard deviation.

**Table 2:** Comparison of candidate K-means solutions.

Age Stratum	k	R^2^	AIC	BIC	Silhouette	CH	Cluster Sizes
6–12 years	2	0.632	613.4	645.4	0.520	696.0	148/259
6–12 years	3	0.822	313.1	361.3	0.530	932.6	133/151/123
6–12 years	4	0.844	285.9	350.1	0.440	724.8	76/146/62/123
6–12 years	5	0.863	262.4	342.5	0.370	633.5	70/122/61/79/75
13–19 years	2	0.692	299.4	326.9	0.550	514.5	105/126
13–19 years	3	0.828	182.1	223.4	0.510	549.4	92/76/63
13–19 years	4	0.854	166.1	221.2	0.460	443.4	23/75/74/59
13–19 years	5	0.873	157.0	225.8	0.440	387.8	46/56/32/23/74

Note. AIC, Akaike information criterion; BIC, Bayesian information criterion; CH, Calinski–Harabasz index; R^2^, explained standardized variance. Lower AIC and BIC values indicate better relative fit according to the respective information criterion and are comparable only among candidate solutions fitted within the same age stratum. Candidate solutions were evaluated jointly using information criteria, explained variance, separation indices, group sizes, and clinical coherence. In the 6–12-year stratum, AIC and BIC favored k = 5, whereas silhouette and CH favored k = 3. In the 13–19-year stratum, silhouette favored k = 2, CH favored k = 3, BIC favored k = 4, and AIC favored k = 5. The retained three-group solutions should therefore be interpreted as balanced exploratory representations rather than as uniquely optimal partitions.

**Table 3:** Observed clinical characteristics of the retained exploratory burden groups.

Age	Burden Group	*n* (%)	DMF-T, Mean ± SD	PI, Mean ± SD	GI, Mean ± SD	CPI 0/1/2, *n* (%)
6–12	Low	123 (30.2)	1.80 ± 1.21	0.82 ± 0.22	0.48 ± 0.19	113 (91.9)/10 (8.1)/0 (0.0)
6–12	Intermediate	151 (37.1)	4.78 ± 1.28	1.74 ± 0.28	1.34 ± 0.29	14 (9.3)/123 (81.5)/14 (9.3)
6–12	High	133 (32.7)	7.32 ± 1.42	2.70 ± 0.24	2.17 ± 0.28	0 (0.0)/23 (17.3)/110 (82.7)
13–19	Low	76 (32.9)	2.05 ± 1.21	0.81 ± 0.24	0.54 ± 0.23	70 (92.1)/6 (7.9)/0 (0.0)
13–19	Intermediate	63 (27.3)	4.97 ± 1.37	1.79 ± 0.28	1.29 ± 0.27	6 (9.5)/47 (74.6)/10 (15.9)
13–19	High	92 (39.8)	7.28 ± 1.36	2.71 ± 0.24	2.14 ± 0.25	0 (0.0)/19 (20.7)/73 (79.3)

Note. CPI is ordinal. The full CPI 0/1/2 distribution is shown because categorical frequencies are clinically more interpretable than a mean for this measure. DMF-T denotes permanent-dentition caries experience assessed exclusively in fully erupted permanent teeth in both age strata.

**Table 4:** Demographic characteristics of the retained burden groups.

Age	Burden Group	Age, Mean ± SD	Girls, *n* (%)	Urban, *n* (%)	Dolj/Gorj/Olt
6–12	Low	9.59 ± 1.86	73 (59.3)	78 (63.4)	39/43/41
6–12	Intermediate	9.62 ± 1.76	81 (53.6)	87 (57.6)	50/58/43
6–12	High	9.70 ± 1.76	64 (48.1)	80 (60.2)	42/45/46
13–19	Low	14.76 ± 1.51	36 (47.4)	38 (50.0)	23/21/32
13–19	Intermediate	14.81 ± 1.53	38 (60.3)	37 (58.7)	22/19/22
13–19	High	14.42 ± 1.51	45 (48.9)	53 (57.6)	37/27/28

**Table 5:** Agreement between Ward hierarchical and K-means classifications.

Stratum	*n*	Ward R^2^	Ward Silhouette	Ward Sizes	Matched Labels	Cramer’s V
6–12 years	407	0.818	0.520	128/151/128	395/407 (97.1%)	0.957
13–19 years	231	0.827	0.510	90/76/65	227/231 (98.3%)	0.973

Note. Ward groups were matched to K-means groups according to the similarity of their standardized cluster centers before participant-level agreement was calculated. The reported agreement represents concordance between two algorithms applied to the same standardized variables and participants; it is not a measure of bootstrap stability or external reproducibility.

## Data Availability

De-identified data may be made available by the corresponding author on reasonable request, subject to institutional, ethical, and data-protection requirements. This secondary analysis uses the same 638-participant clinical database as a companion manuscript examining age-stratified associations between CPI categories and permanent-dentition DMF-T. Recruitment procedures, eligibility criteria, clinical measurements, examiner calibration, and several descriptive characteristics are necessarily shared. The companion manuscript evaluates bivariate and adjusted CPI–DMF-T associations using correlation analyses, group comparisons, and Poisson regression models, whereas the present manuscript examines the joint multivariable burden structure of DMF-T, PI, GI, and CPI using exploratory cluster analysis. The companion manuscript and its current submission status are disclosed in the cover letter and will be supplied to the editors to permit assessment of the degree of overlap and the incremental contribution of the present analysis.

## Supplementary Materials

The following supporting information can be downloaded at: https://www.mdpi.com/article/10.3390/medicina62091654/s1, Figure S1. Analytical workflow used in the revised age-stratified exploratory cluster analysis; Table S1. Dimensionality analysis and correspondence with the composite burden score; Table S2. Sensitivity of the retained burden structure to CPI handling and distance framework; Table S2a. Clinical characteristics of Gower-PAM clusters; Table S3. Alternative two-cluster solution in participants aged 13–19 years; Table S3a. Cross-classification of the adolescent k = 3 and k = 2 solutions; Table S4. Repeated 80% subsampling stability of the retained k = 3 K-means solutions.
