# Supplementary material for: Exploratory Grouping Along a Shared Oral-Health Burden Continuum in Children and Adolescents: An Age-Stratified Cluster Analysis
Source: Medicina (Kaunas). 2026 Aug 28;62(9):1654. doi: 10.3390/medicina62091654 (PMC13609836; doi:10.3390/medicina62091654)
Supplement: Supplementary file 1 [file medicina-62-01654-s001.zip › medicina-4481242-supplementary.pdf]

Supplementary Material

Exploratory Grouping Along a Shared Oral-Health Burden Continuum in Children and Adolescents: An Age–Stratified Cluster Analysis

All analyses below are revision-stage sensitivity or robustness analyses performed on the same complete-case age-stratified datasets used in the main manuscript. Cluster labels were aligned according to the low–intermediate–high burden ordering where required because numerical cluster labels are arbitrary.

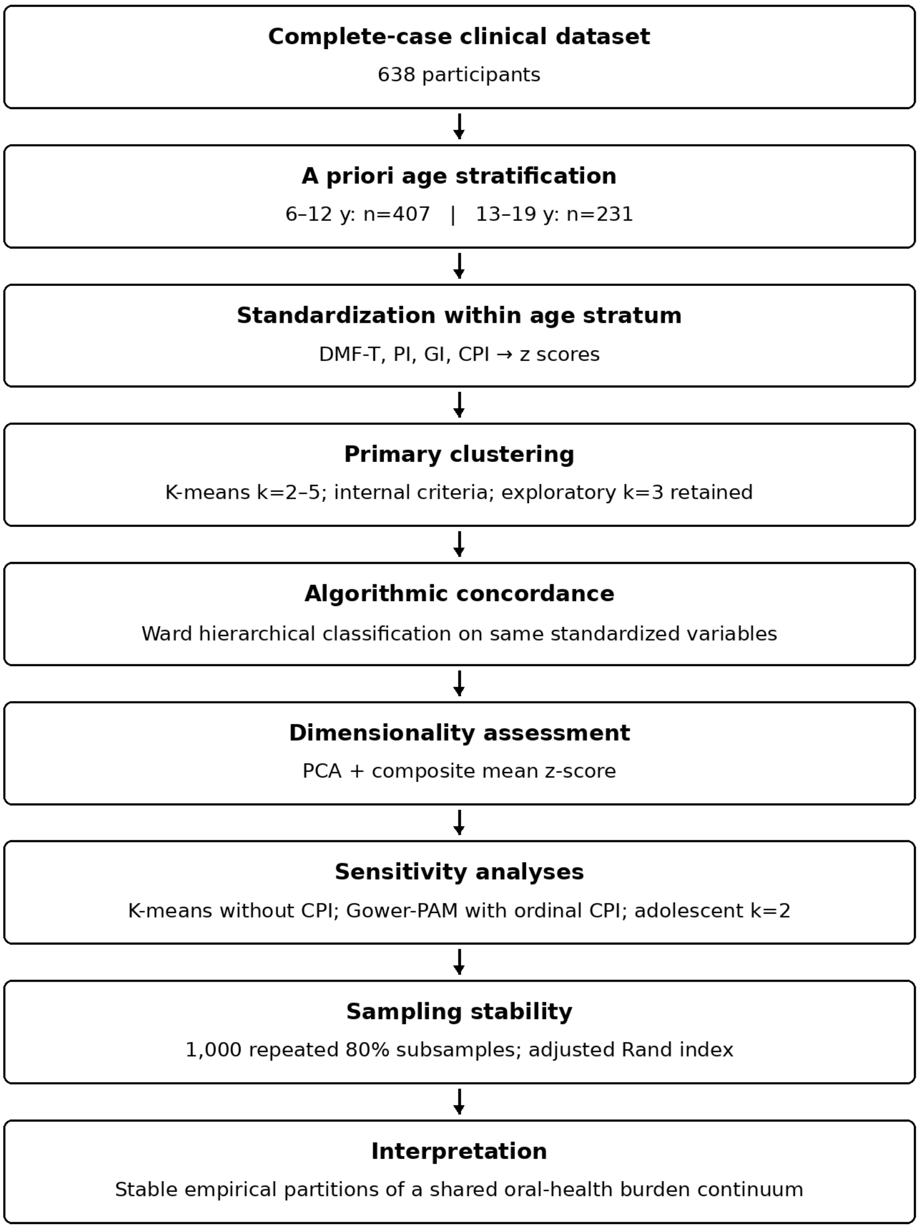

Figure S1. Analytical workflow used in the revised age-stratified exploratory cluster analysis.

Table S1. Dimensionality analysis and correspondence with the composite burden score

Age stratum	PC1 eigenvalue	Variance explained by PC1	DMF-T loading	PI loading	GI loading	CPI loading	Spearman ρ: composite score vs ordered cluster
6–12 years	3.640	91.0%	0.924	0.975	0.959	0.958	0.942 (p < 0.001)
13–19 years	3.672	91.8%	0.930	0.977	0.962	0.962	0.939 (p < 0.001)

Parallel analysis retained one principal component in each age stratum. The composite burden score was the arithmetic mean of standardized DMF-T, PI, GI, and CPI values. Ordered cluster membership was coded 1 = low, 2 = intermediate, and 3 = high burden.

Table S2. Sensitivity of the retained burden structure to CPI handling and distance framework

Age stratum	Sensitivity analysis	Alternative cluster sizes	Silhouette	Participant agreement with original K-means	Cramer's V	Adjusted Rand index
6–12 years	K-means excluding CPI	129 / 130 / 148	0.540	392/407 (96.3%)	0.947	—
13–19 years	K-means excluding CPI	94 / 77 / 60	0.520	222/231 (96.1%)	0.939	—
6–12 years	Gower distance + PAM, CPI ordinal	124 / 156 / 127	0.601	396/407 (97.3%)	—	0.918
13–19 years	Gower distance + PAM, CPI ordinal	75 / 64 / 92 (low/intermediate/high)	0.578	220/231 (95.2%)	—	0.866

For the no-CPI K-means sensitivity analysis, the alternative numerical labels were aligned to the original low–intermediate–high burden ordering before agreement was calculated. For Gower-PAM, CPI was explicitly treated as an ordered factor. Reclassifications were predominantly between adjacent burden levels.

Table S2a. Clinical characteristics of Gower-PAM clusters

Age stratum	Gower-PAM burden group	n	Mean DMF-T	Mean PI	Mean GI	CPI 0 / 1 / 2, n (%)
6–12	Low	124	1.831	0.830	0.487	115 (92.7) / 9 (7.3) / 0 (0.0)
6–12	Intermediate	156	4.827	1.774	1.370	12 (7.7) / 131 (84.0) / 13 (8.3)
6–12	High	127	7.370	2.702	2.169	0 (0.0) / 16 (12.6) / 111 (87.4)
13–19	Low	75	2.040	0.807	0.534	70 (93.3) / 5 (6.7) / 0 (0.0)

13–19	Intermediate	64	4.969	1.845	1.326	6 (9.4) / 53 (82.8) / 5 (7.8)
13–19	High	92	7.261	2.665	2.112	0 (0.0) / 14 (15.2) / 78 (84.8)

Table S3. Alternative two-cluster solution in participants aged 13–19 years

The k = 2 solution had $R^2 = 0.692$, silhouette = 0.550, AIC = 299.4, BIC = 326.9, and Calinski–Harabasz index = 514.5. Cluster 1 represents the lower-burden group and Cluster 2 the higher-burden group.

Characteristic	Lower-burden k=2 (n=105)	Higher-burden k=2 (n=126)
Standardized center: DMF-T	−0.914	0.762
Standardized center: PI	−0.943	0.786
Standardized center: GI	−0.906	0.755
Standardized center: CPI	−0.873	0.728
Observed DMF-T, mean ± SD	2.571 ± 1.427	6.897 ± 1.413
Observed PI, mean ± SD	1.036 ± 0.434	2.500 ± 0.426
Observed GI, mean ± SD	0.723 ± 0.379	1.931 ± 0.431
CPI 0 / 1 / 2, n (%)	74 (70.5) / 30 (28.6) / 1 (1.0)	2 (1.6) / 42 (33.3) / 82 (65.1)
Age, years, mean ± SD	14.74 ± 1.53	14.56 ± 1.52
Girls, n (%)	51 (48.6)	68 (54.0)
Urban residence, n (%)	56 (53.3)	72 (57.1)
Dolj / Gorj / Olt, n	30 / 30 / 45	52 / 37 / 37

Exploratory demographic comparisons for the k = 2 solution were non-significant for sex ($\chi^2 = 0.668$, $p = 0.414$; Cramer's V = 0.054) and residence ($\chi^2 = 0.336$, $p = 0.562$; Cramer's V = 0.038). County showed $\chi^2 = 5.551$, $p = 0.062$; Cramer's V = 0.155.

Table S3a. Cross-classification of the adolescent k = 3 and k = 2 solutions

Original k=3 burden group	Lower-burden k=2	Higher-burden k=2	Total
Low	76	0	76
Intermediate	29	34	63
High	0	92	92
Total	105	126	231

The original low and high groups were completely preserved at the extremes of the k = 2 solution, while the intermediate k = 3 group divided 46.0%/54.0% between the broader lower- and higher-burden partitions.

Table S4. Repeated 80% subsampling stability of the retained k = 3 K-means solutions

Metric	6–12 years	13–19 years
Reference cluster sizes	123 / 133 / 151	63 / 92 / 76
Repetitions	1,000	1,000
Subsample proportion	80%	80%
Mean ARI	0.9958	0.9873
Median ARI	1.0000	0.9854
SD ARI	0.0050	0.0134
IQR ARI	0.0096	0.0297
2.5th–97.5th percentile	0.9901–1.0000	0.9683–1.0000
Minimum–maximum ARI	0.9715–1.0000	0.9259–1.0000
Replicates with ARI ≥ 0.80	100%	100%
Replicates with ARI ≥ 0.90	100%	100%

Reference cluster sizes are reported in the internal numerical cluster-label order of the reference K-means solutions, rather than in the aligned low–intermediate–high burden order; numerical cluster labels are arbitrary and do not affect ARI.

Reference partitions were reproduced using Hartigan–Wong K-means with random seed 1, 100 random starts, and iter.max = 100. Each 80% subsample was drawn without replacement and refitted with 50 random starts. ARI compares the subsample partition with the reference labels restricted to the sampled participants.
